# How can policies impact the relational process of deprescription? A realist review protocol with an initial theory

**DOI:** 10.3389/fpubh.2025.1536147

**Published:** 2025-04-16

**Authors:** Jean Macq, María López-Toribio, Anne Spinewine

**Affiliations:** ^1^Institute of Health and Society (IRSS), Université catholique de Louvain, Brussels, Belgium; ^2^Clinical Pharmacy and Pharmacoepidemiology Research Group (CLIP), Louvain Drug Research Institute (LDRI), Université catholique de Louvain, Brussels, Belgium

**Keywords:** deprescription, policies, realist review, protocol, initial theory

## Abstract

**Introduction:**

Reducing low-value care by deprescribing is a priority. However, current policies often fail due to healthcare provider resistance, lack of incentives, and poor patient engagement. We believe this is due to insufficient focus on the relationships between key agents in the deprescribing process, making ‘one-size-fits-all’ interventions ineffective. Our goal is to understand ‘how, why, for whom, and in what contexts’ policies affect large-scale deprescribing.

**Methods:**

To achieve our research aim, we propose an inductive an abductive and retroductive phases. The inductive phase involved an exploratory review of the literature, leading to the development of an initial theory presented in this article. The initial theory includes three interconnected hypotheses: (a) the success of deprescription depends on the behaviours and interactions of specific agents within the deprescription process. (b) These interactions are influenced by power and trust dynamics, creating either a vicious or virtuous causal loop. (c) The impact of policy on the deprescription chain depends on its ability to address the diversity of the deprescription process and the agents within a given territory, considering dimensionality, ruggedness, and context-specificity. The abductive phase will test the initial theory’s applicability, while the retroductive phase will propose new mechanisms to update it. This forms the core of our realist literature review. After screening, selected articles will assess how well the initial theory explains policy impacts on the deprescription process in specific context. Our article analysis will combine semantic and latent content analysis for the abductive phase, and retroductive analysis. Finally, we will compare the updated theory with existing frameworks.

**Discussion:**

The ultimate purpose of this study is to guide policymakers by providing a framework for informed decision-making before implementing large-scale deprescription policies. Its originality lies in emphasizing the relational nature of the deprescription process and examining policy implementation through the lens of complexity.

## Highlights

- Shared decision-making between patients and healthcare providers is at the core of deprescription process.
- Selecting the appropriate healthcare provider for deprescription should be based on trusting relation and power to conduce deprescription.
- Implementing a brokering strategy or agent to contextualize policies for deprescribing is important for scaling-up.
- Identify conducive forms of governance at organizational, interorganizational, or territorial (place-based) levels is a condition for local adaptation.

## Introduction

Promoting high-value care has been a key focus of health system reform, including reducing unnecessary or harmful care. In pharmaceuticals, this involves addressing potentially inappropriate use and the need for deprescribing. The deprescription process is the set of activities aimed at safely and effectively stopping harmful medications (1), “within the context of an individual patient’s care goals, current level of functioning, life expectancy, values, and preferences” (2). Several interventions have been tested or implemented to increase implementation of deprescription activities. This includes guidelines, new task distribution between healthcare providers (e.g., between GP and pharmacist), funding healthcare providers for part of deprescription chain (mainly medication review), monitoring (de)prescription, payment method [e.g., Pay for Quality (P4Q) initiatives] to engage providers in deprescription, restriction in reimbursement of drugs, quality measurement programs to incentivise primary care providers to review or reduce medications, patient education to enhance Shared Decision Making (SDM), and gradual reduction of medication in residential facilities (mainly in nursing homes) (3, 4).

### Deprescription as part of a complex chain of care: the relational aspect between key agents at the centre of the process

Many trials have tested interventions to stop harmful medications. A recent realist review identified key mechanisms for successful deprescription: Shared Decision Making (SDM), multidisciplinary work, continuity of care (relational, informational, and management), and monitoring (5). This has been further supported by recent research which aimed to describe care trajectories relevant to BSH deprescribing initiated at the hospital level in 6 European countries (6).

These mechanisms support the assumption that th*e relation between key agents (healthcare providers and patient) is central to the success of the deprescription process.* Relational factors can influence various stages of the deprescription process, including the sequence of medication history, identification of potentially inappropriate prescription, determining whether the potentially inappropriate medication can be discontinued, planning the withdrawal regimen, followed by support and monitoring during the tapering process (1). Central to the relational nature of the deprescription process, is the SDM, as indeed deprescription depends, above all, on the informed decision of the patient and informal caregivers.

### Policies to support large scale implementation of deprescription within a territory

Large-scale implementation of the deprescription process can be enhanced by introducing policies. *Policies* are decisions made for a territory (country, region, or local system) and operationalized through various *levers* such as legislation, financial incentives, resource allocation, guidelines, clinical decision supports, norms, and goal setting. These levers aim to enhance implementation of *programs or strategies* to favour deprescription at the *(inter)organizational or individual level*.

Translating policies into effective deprescription programs faces challenges, including difficulties in scaling up successful interventions (*bottom-up approach*) and unexpected policy outcomes (*top-down approach*). Firstly, scaling-up issues often stem from stakeholder acceptance and the dynamics of their networks at political or operational levels within specific health systems (7). This could be the case for mandatory strategies such as legislation (e.g., change in reimbursement, initiation of public reporting of antipsychotic use) (8). The same stands for prescription monitoring policies; pay-for-performance incentives to prescribers; driving safety policies or educational campaigns promoting non-drug alternatives. Secondly, unexpected results may include long-term effects that are different from short-term effects. For example, a review of the outcomes of different policies to reduce the use of Benzodiazepine receptor agonists (BZRA) among older adults found that prescription monitoring policies led to the highest rate of discontinuation but triggered inappropriate substitutions (7).

We assume that these challenges can be better managed if policy design focuses on the relational dimension between key agents in the deprescription chain (3). Considering this may also strengthen the robustness of the policy, i.e., “the capacity to maintain over time despite contingent or critical fluctuations arising from external changes or internal challenges, the specific functions/goals of a policy—in terms of behaviour to be regulated and values to be delivered” (9).

As a result of this assumption, it is important to consider complexity properties when studying policies, such as context-sensitivity, ruggedness, and dimensionality (10). First, the capacity of policy decisions to act at a large scale is likely to be context-sensitive. As a consequence, policy consequences may vary based on context (10). Secondly, the policy landscape may be described through its ruggedness, i.e., the interdependence between policies making that the effectiveness of any one decision cannot be determined in isolation because it depends on what other (policy) decisions are made (10). Finally, the focus on relational aspects of the deprescription chain suggests that policy decisions, leverages, and programs should give specific attention to the behaviour of agents involved in the deprescription chain and their interaction. This is referred to as the high dimensionality of the program that policy aim to influence, i.e., multiple coordinated actions across multiple interacting agents (10).

### Aims and objectives

The objective of this research is to better understand *‘how, why, for whom, and in what contexts’* policies impact the implementation of the deprescription process at a large scale and how robust it is.

As explained above, we make the assumption that the relation between key agents is central to the process of deprescription. Also, we will consider the influence of policy on these relational characteristics through a sequence of policy decisions, operationalized through leverages, acting on key agents for programs and strategies to enhance the deprescription process.

As a consequence, we will develop our understanding of policies through two questions:

- How, why, and for whom specific interventions that have shown their potential in acting on key deprescription actors’ relationships may be enhanced by adequate policy landscape in a specific context? This question will address the contribution of policies in the effective diffusion of innovative intervention in territories through a bottom-up process.
- How, why, and for whom policy implementation may act on relationships between key actors and therefore lead to a large-scale deprescription process? This question will address the contribution of policies to deprescription.

These questions find their justification in key findings from recent reviews is presented in Table 1, justifying hypothesis that will be explored in the realist review.

**Table 1:** Key findings from key review articles.

Key findings from recent reviews	Hypothesis to be explored in realist review
Gap of evidence to design policies in relation to patient and/or prescriber experiences of specific interventions to aid deprescribing medication (20)	The relational dimension between key agents of the chain of deprescription may be a key determinant of the acceptance of specific policies and/or interventions
Moderate effectiveness of educational programs that target the public, patients and health professionals to encourage use of non-drug substitutes, at least in the short term (7)	Educational and awareness programs effectiveness is highly dependent of the credibility of and the relation between the targeted patient or the health professional and the agent who carry the program.
Absence of effectiveness financial deterrents through insurance scheme delisting or financial incentivization in the form of a pay-for-performance supplement to prescribers (7)	Policies focusing on financial deterrents or incentive may not take enough in consideration the relational dimension between key agents of the chain of deprescription
Inconsistent policies across jurisdictions, limited support for deprescribing interventions (3). For example monitoring as a strategy to reduce opioid has shown mixed effects in different jurisdictions in the US (7)	It is important to consider contextual elements to adapt policies to realities of different juridictions. This includes policy landscape, drug availability, role played by different healthcare providers in Deprescription
Unintended effects – for instance, inappropriate substitution following policies focusing on specific potentially inappropriate medication (3, 7)	

In the next sections, we will first explain the methodology, followed by some points of discussion and conclusions.

## Methodology

To reach the aim of our research, we will perform a realist review. “*Realist reviews are explanatory and strive to unpack ‘how, why, for whom, and in what contexts’ policies and programmes work or do not work*.” “*This is done by theorising on the underlying mechanisms that may explain why and how change occurs*” (11).

We justify that methodological choice by the complexity of the process of deprescription. For such a topic, we need to go beyond the simple outcome evaluation and “copy-paste” mode of scaling up [which is called “evidence-based intervention” in the work of Ornstein et al. (10)].

We will follow the usual reasoning steps of realist review, although adopting an iterative approach in our review. This will include an inductive phase, an abductive and retroductive reasoning.

The aim of the inductive phase is to propose an initial theory. This is followed by an abductive phase to delimit the application of an initial theory (by specifying the type of context and of process of de-prescription to which it applies) and operationalizing it (anchoring concretely the theory in specific contexts). Finally, the retroductive phase is a form of inductive approach (i.e., “*moving from a surface phenomenon to a deeper understanding*”) (11). It aims at identifying changes that could not be explained by the initial theory, propose possible new mechanisms and updating the initial theory.

The overall methodological process is synthesized in Table 2 hereafter. It is explained in details in the following sections.

**Table 2:** Steps in the methodology.

Step of the realist review	Purpose	Details of the step
Inductive phase	Propose an initial theory	Exploratory literature review Stakeholders consultation
Screening and selection of articles	Identify policies or interventions that have been evaluated	Screening of databases Selection of articles
Analysis – semantic analysis	Initial description of the policy, intervention, context and evaluation method	Coding of population, intervention, context and type of study in NVivo
Analysis – abductive phase	Test the applicability of the theory	Coding, based on key concepts of the initial theory
Analysis – retroductive phase	Identify new mechanisms to eventually update theory	Synthetic note for each article to exchange intuitions between researchers Updated coding in addition to the coding from the abductive phase Updated mechanisms to update initial theory
Comparison with existing frameworks and review article that analyse interventions and policies aiming at deprescription	Identify what the updated theory adds	Coding of review and frameworks based on key concepts of the updated theory Identification of addition to existing frameworks

### The inductive phase to propose an initial theory: trust and power between agents as a glue to allow for effective deprescription

The initial theory was developed through an exploratory and interpretive literature review, primarily based on two realist reviews on deprescription (3) and inter-organisational collaboration (12). Additionally, this theory builds on concepts of trust and power. It was further developed by testing it in Belgian deprescription policies with key stakeholders.

As explained before, we took the perspective of deprescription as complex process, influenced by the relations between different agents in a territory where policy is being implemented. Three levels of hypothesis are proposed. Firstly, the success of deprescription depends on each agent’s behaviour and their interactions within the care process. Secondly, these interactions are influenced by power and trust dynamics, creating either a vicious or virtuous causal loop. Thirdly, the policy impact on the deprescription chain depends on its ability to address the diversity of the care process and agents in a territory, enhanced by considering complexity dimensions: dimensionality, ruggedness, and context-specificity.

To start, deprescription involves a sequence of activities, including reviewing medication history, identifying inappropriate prescriptions, deciding on cessation, planning withdrawal, and providing support and monitoring during tapering. The success of these activities depends on specific agents’ behaviours, which vary by context. For example, in ambulatory settings, interactions between patients, pharmacists, and GPs differ from those in hospitals or nursing homes. This forms the basis of our initial theory.

Our theory starts with the idea that the attributes of specific agents and their interactions are crucial for achieving optimal behaviours in the care process. This leads to effective deprescription and a strengthened health system.

The main agents in the care chain include patients (taking inappropriate medication), informal caregivers, GPs, pharmacists, specialist MDs (e.g., internal medicine specialists, geriatricians, neurologists, psychiatrists, and pneumologists), and nurses. They are characterized by their roles in deprescription activities. Key considerations include how tasks and responsibilities are distributed among professionals, specifically task sharing, transfer, or delegation. This is detailed in the next part of our theory, which specifies classes of agents.

We propose grouping the above-mentioned agents into different functions to ensure a successful deprescription process:

- beneficiary agent (the patient)
- responsible agent (the one acting in the deprescription)
- accountable agent (the one taking the overall responsibility for the action)
- collaborative agent (the one with whom responsible and beneficiary agents may need to collaborate to ensure a seamless chain of care)

According to the different stages of the deprescription process, the same agent can be responsible, accountable or collaborative.

The different groups of agents, defined by their function, may have different expected behaviours, influenced by possible mechanisms (Table 3). We drew inspiration from Reeve (5) and Aunger (13).

**Table 3:** Examples of functions, agents, expected behaviours and mechanisms that could influence such a behaviour.

Function	Example of agent	Example of expected behaviour (or role)	Example mechanism that could influence expected behaviour
Beneficiary agent (the patient)	An older person taking BZRA People taking PPI People taking opioid / Narcotics or Gabapentinoids medications for chronic pain^a^	Decide deprescription Follows tapering plan Accept living without medication	Readiness to make medication change Faith in non-pharmacological therapies Expected impact on quality of life
Responsible agent (the one acting in the deprescription)	GP Pharmacist …	Medication review Decide deprescription Tapering process Monitoring and follow-up	Feel able to take time and headspace Reluctance to make medication change because of the difficulty to justify it to the other agents
Collaborative agent	Nurse Internal medicine specialist or geriatrician	Perform complementary actions to those performed by the responsible agent in the chain of care Transmit necessary information to the responsible agent when necessary	Raise confidence in managerial continuity
Accountable agent	GP Specialist MD …	Give permission to other providers to act	Assume the final responsibility in part or in the whole process, even if they do not act concretely in deprescription.

a https://www.deprescribingnetwork.ca/useful-resources/.

Agent characteristics are influenced by interactions with other agents, including how information is shared. A key interaction is the decision-making process about deprescription between the patient (*beneficiary agent*) and care providers (*responsible agents*), such as GPs, pharmacists, and specialists. This interaction depends on the type of interprofessional collaboration during the deprescription process. Other agents are important due to their influence on the characteristics and interactions of responsible and beneficiary agents.

The capacity of beneficiary and responsible agents to act in deprescription can be conceptualized as their ‘power,’ as proposed by Bourdieu. According to Bourdieu, power derives from the possession of capital (economic, cultural, social, and symbolic resources) and is ‘field-specific’ (related to a specific domain) (12). If an agent lacks sufficient capital to perform an activity, they may be vulnerable. This is often the case when deprescription is avoided due to uncertainties about patients’ or other providers’ reactions. Such uncertainty can prevent a responsible agent from feeling able to take the time and mental space to make a justifiable decision, leading to reluctance in proposing deprescription.

In this context (of complex task performance), trusting others is recognized as a crucial mechanism. We define trusting others as “the willingness to accept the risk of making oneself vulnerable to another person or party” (14). The issue of trust is also at stake with mechanisms already mentioned above such as fearing negative consequences, feeling supported, getting permission, having clear responsibilities and task allocation, and sharing information with others (see Table 3).

We can, therefore, make the following proposition that could be considered as one of the final outcomes of a policy on deprescription:

If the patient (beneficiary agent) has confidence in (a) non-pharmacological therapies (e.g., Cognitive Behavioural Therapy for Insomnia), (b) the belief that stopping medication will not affect their quality of life, and (c) the understanding that continuing medication increases the risk of adverse events, then they will be ready to decide on deprescription. This involves following a tapering plan and accepting life without medication. For this to work, the patient must have the ‘power’ to decide and act, which can be facilitated by trust in a specific provider (or group of providers), social support, and broader life conditions.

In his turn, the responsible agent needs to meet some conditions so that he or she can play his or her part. This could be expressed as follows:

For the responsible agent to play their role effectively, they must: (a) feel ownership of the deprescription process; (b) not fear negative consequences; (c) understand the patient’s history and reality; (c) feel supported by the system (other agents and the wider context).

This will be strengthened if the responsible agent can partly anticipate the patient’s (beneficiary agent’s) behaviour.

**Figure 1:**
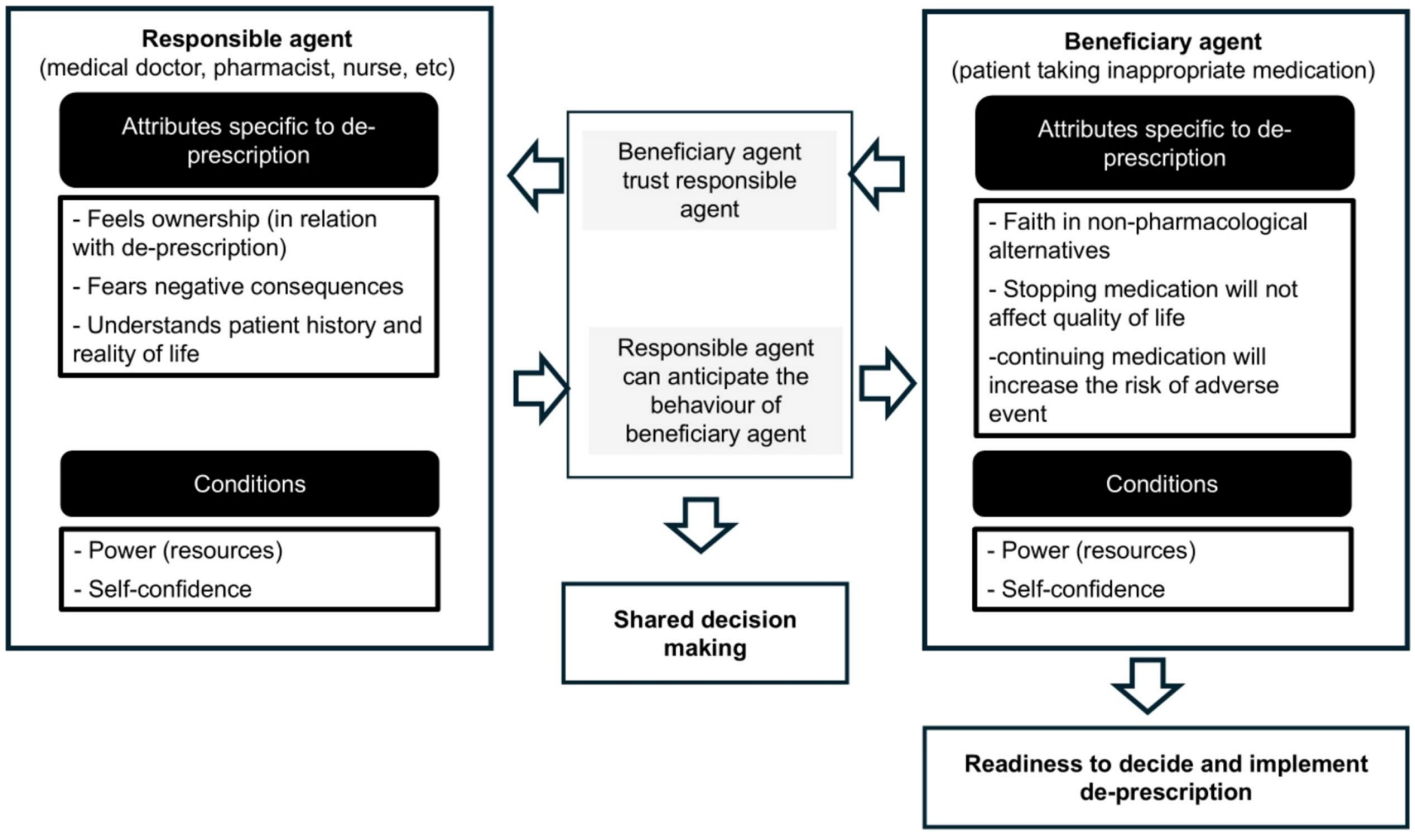
Characteristics and interaction between the two key agents of the deprescription process.

All of this is synthesised in Figure 1 here with hypothetic causal reasoning (arrows). It is largely inspired by Reeve et al. (5).

The choice of strategies or programs at the individual, and inter-organisational level will influence the interplay of power, trust and self-confidence. This can create either a vicious or virtuous circle, depending on the mechanisms triggered by a specific strategy or program.

Without adequate strategies or programs, a vicious circle might be unclenched. This can be presented as follows:

Many responsible agents lack power (are vulnerable) due to the unpredictable outcomes of their actions. They fear being blamed if deprescription fails, leading to a loss of self-confidence. This can result in further loss of power, transferring responsibility to others, or ignoring the need for deprescription.

They may also develop inertia to change and avoid involvement in deprescription.

We hypothesize that trust can break the vicious cycle. In organizational management, two types of trust can be triggered: interactional (relational) and institutional (structural). These forms of trust operate at the individual, team, and organizational levels. Trust-based leadership is expected to activate these types of trust at various levels (14).

To strengthen interactional trust, strategies can be implemented at various levels. At the individual level, this includes enhancing competencies (e.g., for SDM, planning tapering). At the team level, it involves aligning values (e.g., working as a team with the patient as a member). At the (inter-)organizational level, it includes improving communication, collaboration, and conflict resolution among those involved in the deprescription process (14).

Institutional trust focuses on structures like individual contracts and organizational rules. These structures reduce risks by making agent behaviour more predictable. Strategies to build institutional trust include budget allocation, control rules, decision-power distribution, legal regulations, codes of conduct, formal rules, incentive systems, and organizational structure.

Employee participation in designing these institutional strategies and control mechanisms is crucial for building trust (14).

Building on power and trust, we propose the next component of the theory:

Enhancing shared decision-making between the patient and responsible agent can create a virtuous circle, increasing power and trust for both parties.

To reinforce this virtuous circle, strategies at the individual, team, and (inter-)organizational levels can be implemented:

- Individual Level: Boost individual competencies (interactional trust), enhance value alignment, and promote information sharing and decision-making among agents in the deprescription process.
- Team Level: Structure work through multidisciplinary teams to increase individual power. Trust within the team allows members to share vulnerabilities, better handle uncertainties, and build self-confidence. This is crucial when shared decision-making is challenging, such as in the deprescription process. Collaborative behaviour is essential, and the team’s strength and usefulness depend on its composition and history. Effective synergy arises from the combination of skills, knowledge, and resources among team members.

Implementing these strategies fosters a supportive environment that enhances power and trust (13). The history of teamwork is crucial. Initially, the belief that collaboration improves deprescribing predicts the time and resources invested in it. If successful, risk-taking drives collaboration (increasing trust) rather than competition (13, 14).

Finally, strategies targeting organizations or networks can increase trust by:

- Organizing information sharing to ensure managerial continuity
- Clarifying responsibilities and task allocation
- Setting the right team and decentralizing decision-making at the department or team level, while strengthening leadership
- Aligning incentives and budgets to enhance effectiveness and managerial continuity across the deprescription process

These strategies are crucial for ensuring seamless care between responsible and collaborative agents, both within and between organizations.

Effective policies should not only focus on deprescription but also consider the interplay between trust and power within the deprescription chain of care. This requires strategies and programs at both organizational and inter-organizational levels.

We propose focusing on functions, structures, and processes that can transform a vicious circle into a virtuous one, where power and trust interact to facilitate deprescription. Specific programs and strategies should enhance these elements.

- Function: The “advocacy” or “navigator” role improves relational continuity and decision-making. This role, trusted by other agents, is key for shared decision-making. It can be long-term (primary care providers) or short-term (case managers in crisis situations).
- Structure: Multidisciplinary teams support agents and allow flexible task allocation along the deprescription chain.
- Processes: Information sharing and trust-based leadership enhance continuity, clarify responsibilities, and build trust. Trust-based leadership at the network level (local health systems) influences team and organizational communication.

Policy choices should promote these functions, structures, and processes, building on existing policies (*ruggedness*) to optimize strategies. This approach considers the diversity of agents and interactions (*dimensionality*) and adapts to different contexts (*contextuality*).

Based on the above, we propose the final part of the theory:

We hypothesize that effective policies to enhance deprescription on a large scale should follow these steps:

- Identify key agents (beneficiary, responsible, collaborating, accountable) in the deprescription process.
- Develop programs and strategies that leverage power and trust among these agents (e.g., advocacy roles, team structuring, information systems) to shift from a vicious to a virtuous circle.
- Adjust various levers (legislation, training, payment systems, guidelines) to enhance programs and deprescription, considering dimensionality, ruggedness, and contextuality within the targeted territory.

This initial theory will be tested through literature review. In the next section, we explain how screening, selection of article and analysis will be carried out.

### Screening articles

Pubmed and Google Scholar will be used to screen for relevant articles. These databases were chosen opportunistically. Pubmed includes more than 37 million references from MEDLINE and other additional citations. This is supposed to include medical references published in journals with impact factor. Google Scholar was chosen for its ability to include grey literature in addition to publications in journals with impact factor. Published screening strategies targeting deprescription will be used. Namely, we will develop our strategy (see Box 1), based on the equation developed by Shaw et al. (7) and by Morel et al. (15).

BOX 1 **Equation for publication databases screening.**

Screening equations chosen for

In Pubmed we will use the following equations

- ((policy making[MeSH Terms]) OR (analysis, policy[MeSH Terms]) OR (health policy[MeSH Terms]) OR (policies[MeSH Terms]) OR (policy[MeSH Terms])) AND ((deprescriptions* [MeSH Terms]) OR (Drug Tapering [MeSH Terms]) OR “drug discontinu*” OR deprescription OR tapering)
- (“legislation” OR “policy” OR “public policy” OR “health policy” OR “program” OR “patient education” OR “campaign” OR “practice guidelines”) AND ((deprescriptions* [MeSH Terms]) OR (Drug Tapering [MeSH Terms]) OR “drug discontinu*” OR deprescription OR tapering) AND evaluation AND (context OR process)

In Google Scholar, we will use the following equation

- (“legislation” OR “policy” OR “public policy” OR “health policy” OR “program” OR “patient education” OR “campaign” OR “practice guidelines”) AND (“deprescriptions” OR “drug withdraw*” OR “drug discontinu*”)

### Selection of articles

Screened publications will be reviewed for selection in two steps by two independent reviewers, by using Rayyan.^1^

The criteria used for selection of articles will concern the population, the intervention or phenomena of interest, the context, and the study design. They are presented in the table hereafter.

**Table:** 

The population	Patient taking PIM and/or with polymedication
The Intervention or phenomena of interest	Intervention and policies targeting de-prescription of any type of drugs
Context	Country of study will be USA OR Canada OR Europe.
Comparator	Any
Study design	Evaluation study of a policy or an intervention that may lead to policy OR a proposition of a framework or a theory Evaluation study will include process and implementation, time series, and “effectiveness” studies (RCT, quasi experimental, …). Article protocol will be excluded.

As a result, we expect three types of articles for analysis: (a) those proposing frameworks to analyse policies or programs aiming at enhancing deprescription; (b) the review of policies on deprescription; (c) those reporting an evaluation of deprescribing intervention or policy in a specific setting. The first two types of articles will update the initial theory or confirm its value. The third type will be grouped by setting to develop ‘cases.’ A ‘case’ will be a specific setting (country or region) where the initial theory is tested. This helps assess how well our theory explains ‘*how, why, for whom, and in what contexts*’ policies impact large-scale deprescribing and its robustness. A case study will use various articles to describe relevant contextual elements.

### Analysis of the articles: semantic analysis, abductive, retroductive reasoning and comparison with existing frameworks and reviews

The initial theory will be tested to assess its capacity to explain how policies may impact the implementation of the deprescription process in a country (or a region). This will be done in the four steps described hereafter.

First, we will perform a semantic analysis (focus on what is written in the article). We will search for a *description* of (a) the deprescription process (i.e., *from medication review until follow-up and monitoring*), agents involved, their attributes and behaviours; (b) interventions (programmes or strategies) targeting deprescription and their possible link with function (advocacy – navigator), structure (teams and their constitution) or processes (the way of sharing information and trust-based leadership) and (c) policies at country, region or local system level aiming at enhancing deprescription (i.e., *what is the policy all about and how does it operationalize into a program*?).

The second step will be abductive. Practically, we will perform a latent content analysis (*interpret and propose meanings* of the content of the article). Coding of the article content will therefore be done by using key concepts of the initial theory. Furthermore, to perform that interpretive work, we will search for additional relevant information from the context (other concurrent policies that may affect the implementation of the policy described in the article, demographic information on specific agents, organization and functioning of healthcare…).

The third step will be retroductive. It will build and develop further the latent content analysis through the following steps: (a) for each article, we will write notes with initial thoughts about the adequacy of the initial theory; (b) this note will be used to exchange intuitions between researchers and identify possible discrepancies in the initial theory; (c) this will lead to update the reasoning (theory); (d) and update the codes; (e) these new codes will be used to analyse a new article. These different steps will be used until stability with the updated theory is reached.

The last step of the analysis will aim at identifying what our updated theory adds by confronting it to existing frameworks.

The software NVivo ® will be used along all the steps for this analysis.

## Discussion and conclusion

This article presents the protocol for a realist review, including the initial theory. The final aim is to better understand *‘how, why, for whom, and in what contexts’* policies impact the implementation of the deprescription process at a large scale and how robust it is. This article highlights key elements that we aimed to clarify in our initial theory, justifying the methodological approach we propose for our literature review.

### Linking policies to the deprescription process through a sequence of determinants at different levels

We defined policies as a decision taken for a territory: country, region or local system level. The evaluation of their effect needs close attention to a sequence that connects this decision with final changes at the healthcare level, in our case with the deprescription process (16). Our initial theory proposes reasoning starting with agents’ behaviours and their interactions as proximal determinants of successful deprescription process; strategies or programs implemented at (inter-)organisational level as intermediary determinants; leverages used to operationalize policy decision as distal determinants. By adopting that reasoning, we acknowledge policy as a contributing factor to change in the deprescription process. In such an approach, it is irrelevant to search attribution of a policy to a specific change in deprescription.

### Focus on the relational nature of the deprescription process: the territory targeted by policies as a complex adaptive system

We focus our initial theory on the relational nature of the deprescription process. This has already been recognised specifically for deprescription (5) and more largely for many other healthcare processes (17). For policy making, this call for an in-depth study of agents and their interaction with other agents or their context, and not only at structures. Therefore, although it is possible to identify “rules” that apply to a group of agents, it is also important to recognise the variability in their behaviour. The theoretical concept of a complex adaptive system has already been used to incorporate that in assessing policies or innovations (18).

In our case, we may consider the territory where policy is decided and implemented and where the deprescription process takes place. We would view this territory as a “complex adaptive system” (CAS), i.e., a system made of a set of interacting agents (patients, informal caregivers, medical doctors, nurses, pharmacists, etc.). Agents are free to act in ways that are not always predictable. They are influenced by and influence their environment which is made of agents and other elements. As a consequence, the whole system’s behaviour is different than the sum of the behaviour of each element (10).

### A literature review to propose “lenses” to understand the dynamics of changes in a specific context rather than building the one-size-fits-all evidence-based intervention

Our initial theory justifies the methodological approach that we propose for this literature review. We adopt the position of Lancaster et al. that says that “evidence, interventions and policy are constituted in knowledge-making practices and it acknowledges that the effectiveness of policy decisions and interventions is always situated and emergent” (19).

The final product of proposing a theory is to learn stakeholders involved in policy making to ask the good question and orient them in causal reasoning, as an initial step before deciding leverage, strategies or programs to improve deprescription. This has consequences on the choices that are made for this literature review.

First, it does not aim at the exhaustivity of studies in a given topic, but rather at relevance and richness (11). To test the capacity of our theory (and eventually adapt it), we need to select articles that allow us to assess its usability in given contexts but also that provide insides to update the theory. The relevance and richness are expected to evolve during the review, justifying an iterative or cyclic process in reading and analysing articles.

Second, we attach particular importance to the latent content of articles. Interpretation and proposition of new meanings by the researchers are considered the key to innovative findings.

Finally, even if the results of the literature review whose methodology has been described in this article are still to come, we hope that this article brings already new insights for policymakers to orient their decisions to improve the deprescription process.

### Key policy recommendations that are expected from this literature review

This realist review is expected to assist decision-makers reasoning in adapting policies and interventions to specific contexts. Amongst other, we hope to guide context sensitive policy decision and implementation stakeholders to respond to the following questions:

- What are the conducive contexts for shared decision making between patient and healthcare provider and how could policy enhance it?
- Which healthcare provider should be involved in the deprescription (including general practitioners, pharmacists, nurses, specialists, psychologists, etc.)? For what part of the deprescribing process? Why? How? Under what conditions?
- Which brokering strategy or agent should be put in place to help contextualize policies to support deprescribing? Why? How? Under what conditions?
- Which type of governance should be favoured at organisational, interorganisational level or territorial (place-based) level to favour inter-professional collaboration and facilitate deprescription process? Why? How? Under what conditions?

## Data Availability

The original contributions presented in the study are included in the article/supplementary material, further inquiries can be directed to the corresponding author.

## Footnotes

^1^ https://rayyan.ai/
